# Head Horn Enhances Hydrodynamic Perception in Eyeless Cavefish

**DOI:** 10.1002/advs.202406707

**Published:** 2024-09-23

**Authors:** Zhiqiang Ma, Zheng Gong, Yonggang Jiang, Peng Wu, Changxin You, Zihao Dong, Hongchao Cao, Zhen Yang, Yahui Zhao, Huawei Chen, Deyuan Zhang

**Affiliations:** ^1^ Institute of Bionic and Micro‐Nano Systems School of Mechanical Engineering and Automation Beihang University Beijing 100191 China; ^2^ International Research Institute for Multidisciplinary Science Beihang University Beijing 100191 China; ^3^ Artificial Organ Technology Lab Bio‐manufacturing Research Center School of Mechanical and Electric Engineering Soochow University Suzhou 215021 China; ^4^ Centre for Artificial Intelligence and Robotics Hong Kong Institute of Science & Innovation Chinese Academy of Sciences Hong Kong 999077 China; ^5^ Key Laboratory of Zoological Systematics and Evolution Institute of Zoology Chinese Academy of Sciences Beijing 100101 China

**Keywords:** Chinese cavefish, head horn, hydrodynamic perception, lateral line, sensor arrangement

## Abstract

Fish can use hydrodynamic stimuli, decoded by lateral line systems, to explore the surroundings. Eyeless species of the genus *Sinocyclocheilus* have evolved conspicuous horns on their heads, whereas the specific function of which is still unknown. Meanwhile, the eyeless cavefish exhibits more sophisticated lateral line systems and enhanced behavioral capabilities (for instance rheotaxis), compared with their eyed counterparts. Here, the influence of head horn on the hydrodynamic perception capability is investigated through computational fluid dynamics, particle image velocimetry, and a bioinspired cavefish model integrated with an artificial lateral line system. The results show strong evidence that the head horn structure can enhance the hydrodynamic perception, from aspects of multiple hydrodynamic sensory indicators. It is uncovered as that the head horn renders eyeless cavefish with stronger hydrodynamic stimuli, induced by double‐stagnation points near the head, which are perceived by the strengthened lateral line systems. Furthermore, the eyeless cavefish model has ≈17% higher obstacle recognition accuracy and lower cost (time and sensor number) than eyed cavefish model is conceptually demonstrated, by incorporating with machine learning. This study provides novel insights into form‐function relationships in eyeless cavefish, in addition paves the way for optimizing sensor arrangement in fish robots and underwater vehicles.

## Graphical abstract

Blind Chinese cavefish have evolved a unique head horn structure, which is a convergent feature in the eyesight‐reduced cavefish species. In this work, the structure‐function relationships of this specific head horn structure are systemically investigated, adequately revealing that it can enhance hydrodynamic perception and facilitate underwater obstacles recognition, benefiting eyeless cavefish surviving in rigorous cave environments.

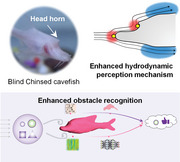

## 1 Introduction

Through the course of evolution, nature has bestowed upon creatures a diverse array of remarkable structures/systems, enabling their survival in harsh and intricate environments.^[^ ^1^ ^]^ In environments where visual information is inaccessible, the perception of flow fields provides animals with an effective strategy for exploring their surroundings. For instance, based on the detected flow information, seals and mosquitoes utilize their whiskers and Johnston's organs to effectively pursue long‐distance prey^[^ ^2^, ^3^ ^]^ and avoid obstacles^[^ ^4^ ^]^, respectively. Delving into the investigation and establishment of the structure‐function relationships not only enhances our understanding of these captivating structures/systems, but also paves the way for the development of bioinspired artificial counterparts applicable in various engineering domains.^[^ ^1^, ^2^, ^3^ ^]^

Over millions of years of evolution, fish and aquatic amphibians have developed an exceptional mechanosensory lateral line system (LLS).^[^ ^5^ ^]^ This system consists of numerous neuromasts, which can be categorized into two types: superficial neuromasts (SNs) and canal neuromasts (CNs).^[^ ^5^ ^]^ Positioned directly on the skin, SNs are exposed to local water movements and exhibit sensitivity to flow velocities as low as 10 µm s^−1^.^[^ ^5^ ^]^ On the other hand, CNs are located within lateral line canals that connect to the surrounding water through a network of pores. CNs are particularly responsive to pressure differences along the lateral line canals, with a minimum threshold of 2 mPa.^[^ ^6^ ^]^ Triggered by hydrodynamic stimuli, the mechanosensory LLS empowers fish to generate a “hydrodynamic imaging” in their brain. This hydrodynamic imaging, often referred to as “touch‐in‐distance,” facilitates the exploration, navigation, and execution of diverse behaviors within their environments.^[^ ^7^, ^8^, ^9^ ^]^ Drawing inspiration from this biological mechanism, engineers have successfully developed impressive artificial lateral line (ALL) systems.^[^ ^9^, ^10^, ^11^, ^12^, ^13^, ^14^, ^15^ ^]^ Nonetheless, the pursuit of biomimetic research persists, aiming to continually optimize engineering designs as an increasing number of exceptional natural structures/systems are unveiled.

Cavefish, despite their loss of visual capacity in completely dark environments, are still able to maintain normal lives. This suggests that in the process of evolution, the absence of vision can be compensated by the development of other sensory capabilities.^[^ ^16^ ^]^ One such compensatory mechanism is the enhancement of the lateral line system, which enables cavefish to rely on hydrodynamic perception capability for exploring their surroundings. Previous studies have provided validation that the eyeless Mexican cavefish is capable of successfully performing a range of daily behaviors solely relying on the assistance of the lateral line system. These behaviors include rheotaxis,^[^ ^8^, ^17^, ^18^ ^]^ obstacle recognition,^[^ ^19^, ^20^ ^]^ shoaling,^[^ ^21^ ^]^ and prey detection.^[^ ^22^ ^]^ These findings highlight the remarkable adaptability and functional efficacy of the lateral line system in compensating for the absence of vision in cave‐dwelling fish.

The genus *Sinocyclocheilus* (Cypriniformes: Cyprinidae), which is endemic to the vast karst region in southwestern China, has gained significant popularity as a cavefish model. This is primarily attributed to its distinction as the largest known radiation of freshwater cavefish worldwide.^[^ ^23^ ^]^ Being well‐adapted to intricate cave environments (Figure S1, Supporting Information), these fish exhibit a range of morphological traits, including eye degeneration, absence of pigmentation, and elongated fins,^[^ ^24^, ^25^ ^]^ which are comparable to those observed in other cavefish species. Furthermore, in addition to the commonly observed morphological adaptations, numerous eyeless species of *Sinocyclocheilus* possess a distinctive and prominent structure known as the humpback or head horn (Figure S2, Supporting Information).^[^ ^26^, ^27^ ^]^ Despite its unique presence, the precise function of the head horn structure in *Sinocyclocheilus* remains unknown. Existing studies have primarily proposed hypotheses regarding its potential roles, such as facilitating fat storage,^[^ ^27^ ^]^ providing brain protection,^[^ ^28^ ^]^ or aiding in acoustic perception.^[^ ^29^ ^]^ However, detailed validation and conclusive evidence supporting these hypotheses are currently lacking. Similar to the Mexican eyeless cavefish, the Chinese eyeless cavefish also exhibits an enhanced lateral line system compared to its surface‐dwelling counterpart.^[^ ^30^ ^]^ Indeed, it is likely that the Chinese eyeless cavefish possesses an improved hydrodynamic perception capability originated from its enhanced lateral line system. This adaptation allows it to detect and interpret hydrodynamic stimuli more effectively, contributing to its overall sensory perception in aquatic environments. For instance, Chen et al discovered that the Chinese eyeless cavefish exhibited enhanced wall‐following behaviors compared to the normal‐eyed species.^[^ ^31^ ^]^ Specifically, the eyeless cavefish demonstrated the highest level of wall‐following behavior, while the normal‐eyed cavefish exhibited the lowest level of such behavior.

As widely acknowledged, the body shape has the potential to modify the surrounding flow fields and may consequently impact the flow sensing capability to a significant extent.^[^ ^19^, ^32^, ^33^ ^]^ For example, Yanagitsuru et al. conducted a study to explore the impact of head width variations on the flow sensing capability of the lateral line system.^[^ ^32^ ^]^ In their research, they developed physical fish head models with different widths (narrow, intermediate, and wide) integrated with pressure sensors to assess their hydrodynamic perception performance. The experimental findings revealed that the anterior region of all model heads exhibited the richest hydrodynamic information. Moreover, the fish head optimized the perception of vortical field by the canal lateral line system, i.e., wide fish heads demonstrated greater sensitivity in perceiving larger vortices, while narrow fish heads exhibited heightened sensitivity to perceiving smaller vortices.

Here, we comprehensively validated that the head horn structure in Chinese eyeless cavefish enhanced hydrodynamic perception by employing multiple and intercalibrated approaches, from aspects of universal flow fields, multiple hydrodynamic sensory indicators, and necessary obstacle recognition behaviors. By leveraging the capabilities of laser scanning and 3D printing techniques, we successfully developed high‐fidelity digital and physical models of cavefish, where the eyeless cavefish species *S. tianlinensis* and the eyed fish species *S. macrophthalmus* were selected as prototypes, allowing us to create accurate representations for further investigation and analysis. Through the analysis of uniform flow fields, it was observed that the head horn shaped eyeless cavefish experienced a stronger hydrodynamic stimulus compared to the conventional streamlined eyed cavefish, by evaluating a series of hydrodynamic sensory indicators, including pressure, pressure gradient, pressure difference, and wall shear stress. Subsequently, the enhanced flow perception strategy (EFPS) utilized by the eyeless cavefish was uncovered as: the head horn body shape rendered it with two stagnation points, and the lateral line system was tightly distributed with these two maximum hydrodynamic stimulus regions. In addition to examining the uniform flow scenario, we further developed a bioinspired cavefish model instrumented with an artificial lateral line system to validate the proposed EFPS in vortical flow fields, due to pressure sensor's strength in capturing spatial perturbations. Furthermore, by integrating machine learning techniques, the cavefish model incorporates the EFPS, demonstrated an enhanced capability for recognizing underwater obstacles. Despite the reducing number of hydrodynamic pressure sensors and the shortening duration of hydrodynamic stimulus, the eyeless cavefish model consistently exhibited higher accuracy in obstacle recognition compared to the eyed cavefish model. We envision that the EFPS employed by the eyeless cavefish can contribute to a better understanding of the functional role of biological head horn structures. Furthermore, this knowledge can be applied to optimize flow field sensing in the field of engineering.

## 2 Results

### 2.1 Influence of Body Shape on the Cephalic Lateral Line System

**Figure 1:**
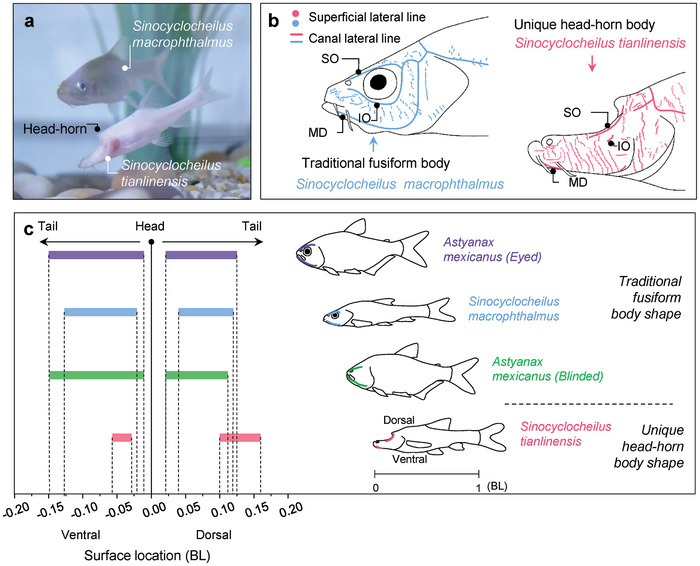
Association between cephalic lateral line system and fish body shape. a) Photograph of the eyeless cavefish *Sinocyclocheilus tianlinensis* with unique head horn body shape and the eyed cavefish *S. macrophthalmus* with traditional fusiform body shape. b) Cephalic superficial neuromasts and canal lines of cavefish species (revised from Ma et al.^[^ ^30^ ^]^ with permission of Wiley.). SO, supraorbital canal; IO, infraorbital canal; MD, mandibular canal. The general courses of the lateral line canals were drawn based on the distribution of canal pores shown in Figure S3 (Supporting Information). c) Surface location of the cephalic lateral line canals (SO and MD) of the four species shown on the right. Data of the Mexican cavefish *Astyanax mexicanus* are taken from Schemmel^[^ ^34^ ^]^ with permission of Springer Nature.

In this study, two species of *Sinocyclocheilus* cavefish, namely *Sinocyclocheilus macrophthalmus* and *Sinocyclocheilus tianlinensis*, were chosen as biological prototypes. As depicted in **Figure** **1**a, the *S. macrophthalmus*, an eyed cavefish, exhibits a fusiform body shape and resides in subterranean rivers near cave mouths or sinkholes. Conversely, the *S. tianlinensis*, an eyeless cavefish, possesses a distinctive head horn body shape and thrives exclusively within deep caves throughout its entire life. As found in Mexican eyeless cavefish, the *S. tianlinensis* evolved enhanced lateral line system for hydrodynamic sensing which compensates the reduced vision capability, compared with eyed *S. macrophthalmus*.^[^ ^30^ ^]^ For instance, in the eyeless *S. tianlinensis*, the SNs are distributed across almost the entire surface of the head, whereas in the eyed *S. macrophthalmus*, they are primarily concentrated in the region around the eye (Figure 1b). Additionally, the cephalic lateral line canal exhibits an intact pattern in the eyed *S. macrophthalmus*, while it displays a reduced and concentrated pattern in the eyeless *S. tianlinensis*. The lateral line canal in *S. tianlinensis* exhibits a stronger constriction structure, enhancing its sensing capabilities compared to that of *S. macrophthalmus*. Interestingly, the reduced lateral line canals in *S. tianlinensis* are predominantly distributed in the region around the snout and head horn (Figure 1b).

Subsequently, in order to investigate the impact of the head horn structure on hydrodynamic sensing performance, we compiled and summarized the characteristic distribution patterns of cephalic lateral line canals in four well‐known cavefish species. As depicted in Figure 1c, the cephalic lateral line canals, specifically the supraorbital canal (SO) and mandibular canal (MD), are situated in the streamlined profile and closely distributed around the snout. However, in the case of the eyeless *S. tianlinensis*, the dorsal MD is concentrated in the region of the head horn. From an evolutionary perspective, we propose that the head horn structure likely contributes to the enhancement of hydrodynamic performance in eyeless cavefish.

### 2.2 Enhanced Uniform Flow Field Perception

**Figure 2:**
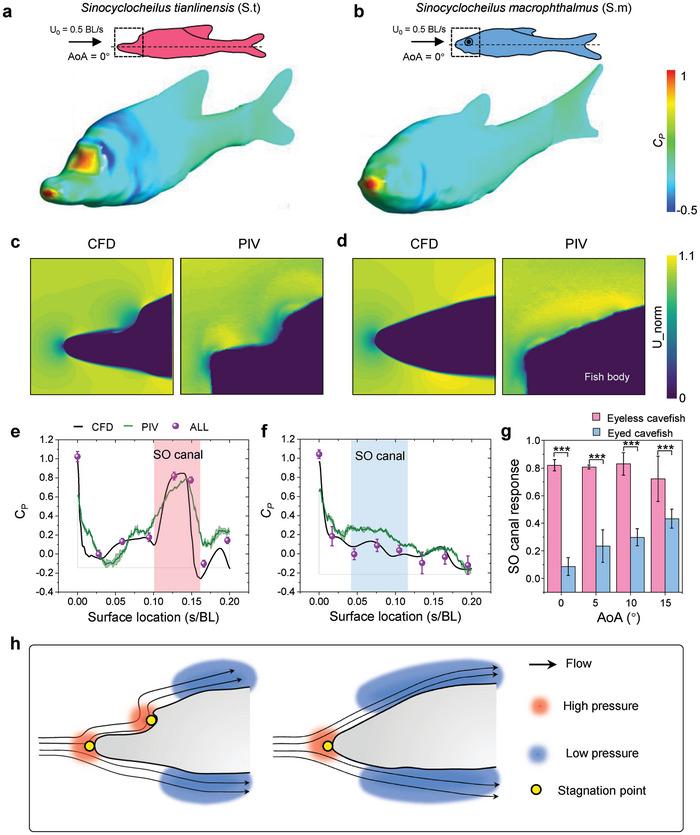
Enhanced flow perception strategy (EFPS) in uniform flow field. a,b): Pressure coefficient (C_P_) distribution on the body surface of the eyeless cavefish *S. tianlinensis* (a) and the eyed cavefish *S. macrophthalmus* (b). Incoming flow velocity U_0_ was 0.5 BL/s (body length/s) and the angle of attack (AOA) was 0°. AOA is defined as the angle between the direction of incoming flow and the longitudinal body axis of the fish. 0°: Flow parallel to the longitudinal body axis of the animal. Black dashed circles indicate high‐pressure regions on the cephalic surface of cavefish. c and d: Flow field distribution around the head in the 0.2 BL region, evaluated via particle image velocimetry (PIV) and computational fluid dynamics (CFD), both in the eyeless c) and eyed d) cavefish. AOA was 0° and the incoming flow velocity was 0.5 BL/s. e and f: C_P_ distribution along the head of the eyeless cavefish *S. tianlinensis* e) and eyed cavefish *S. macrophthalmus* f). Flow velocity was 0.5 BL/s and AOA was 0°. C_P_ was evaluated using CFD, PIV, and ALL systems. g) SO canal response under different AOAs, with a constant incoming flow velocity of 0.5 BL/s. Asterisks indicate statistically significant difference (^∗∗∗^ *P* < 0.001). h) Schematic illustration of the enhanced perception mechanism in the uniform flow field.

3D high‐fidelity digital models of cavefish were obtained through the utilization of laser scanning on biological samples. The hydrodynamic analysis was initially performed through 3D computational fluid dynamics (CFD) simulations. Mimicking gliding motions, the cavefish moved through water at a constant speed of 0.5 BL/s (body length per second) and different angle of attack (AoA) ranging from 0° to 15°. Further information regarding the calculations can be found in the Experiment Section as well as in Figures S4 and S5 (Supporting Information). As anticipated, the CFD simulations indicated that the eyeless cavefish experiences a larger area of high hydrodynamic pressure stimuli compared to the eyed cavefish (**Figure** **2**a,b; Figure S6, Supporting Information). In the case of the eyed cavefish, the high‐pressure area was primarily located at the snout. These findings indicated that the eyeless cavefish experiences high hydrodynamic pressure both at the tip of the snout and in the head horn region. The qualitative presentation of the calculation results suggested that the head horn structure enhances the sensing of hydrodynamic stimuli.

To ascertain the accuracy of simulation results, we utilized particle image velocimetry (PIV) to scrutinize the flow fields encompassing high‐fidelity physical cavefish models, which were fabricated by means of 3D printing digital models (Figure S7, Supporting Information). Under identical working conditions, the acquired results showcased exceptional consistency in the flow field contours surrounding cavefish, as obtained through the utilization of CFD and PIV (Figure 2c,d; Figure S8, Supporting Information). It provided qualitative validation for the accuracy of the theoretical calculation results.

Furthermore, for quantitative assessment of theoretical calculation accuracy, the normalized pressure field was represented using a pressure coefficient (*C_P_*), which was calculated based on the Bernoulli's law, as per the following formulation:

(1) $$C_{P}=\frac{p}{0.5\rho U_{0}^{2}}=1-{\left(\frac{U}{U_{0}}\right)}^{2}$$

where *U_0_* is incoming flow velocity (or the velocity of the swimming fish), *p* is the pressure distribution on the fish's body, *ρ* is water density, and *U* is the local flow speed nearby cavefish head.

The *C_P_* distribution along the cavefish head, as depicted in Figure 2e,f, and Figure S9 (Supporting Information), exhibits a favorable agreement between the measurements obtained through PIV and the calculations derived from CFD. This quantitative validation lent further support to the accuracy of the CFD simulation calculations. The findings revealed that the*C_P_* experienced by eyeless cavefish reaches its maximum at both the snout and head horn regions (Figure 2e). Despite the absence of CNs at the snout tip (Figure 1b), nearby CNs located in the MD displayed sensitivity to pressure variations along the fish's head. Furthermore, a portion of the SO resides within the head horn region (Figure 1b), consequently being exposed to the secondary pressure peak (Figure 2f). In contrast, the eyed cavefish, characterized by a fusiform body shape, exhibited a single pressure peak located on the snout (Figure 2f). Due to the proximity of the canal lateral line system (SO, IO, and MD) to the snout region (Figure 1b), they experienced substantial pressure variations in that specific area. Lastly, the response of the cavefish lateral line system was determined by measuring the maximum hydrodynamic stimuli within the SO canal region. Consistent with expectations, the eyeless cavefish exhibited a higher response in their lateral line system compared to the eyed cavefish (*t*‐test; *P* < 0.001), indicating their heightened sensitivity to hydrodynamic pressure (Figure 2g).

In addition to hydrodynamic pressure, other indicators of hydrodynamic sensing, such as pressure gradients (Figure S10, Supporting Information), pressure difference (Figure S11, Supporting Information), and wall shear stress (Figures S12 and S13, Supporting Information), were assessed to further validate the enhanced function of the head horn structure in hydrodynamic perception. The evaluation of these hydrodynamic sensing indicators collectively demonstrated that the presence of the head horn structure effectively facilitates hydrodynamic perception in the eyeless cavefish.

The flow field surrounding the eyeless cavefish exhibited two distinct stagnation points, where the local flow velocity reached zero. These stagnation points were observed adjacent to both the snout and the head horn regions (Figure 2c). In contrast, the eyed cavefish displayed a flow field with a single stagnation point near the snout (Figure 2d), resembling the flow field observed around a symmetrical airfoil positioned in a uniform flow.^[^ ^19^, ^35^ ^]^ Notably, the eyeless cavefish consistently demonstrated the presence of double stagnation points under various AOAs conditions (Figure S8, Supporting Information). The presence of stagnation points is associated with regions of high pressure, which potentially contribute to hydrodynamic sensing.^[^ ^19^, ^32^, ^33^ ^]^ The high‐pressure region indicates substantial pressure fluctuations, offering valuable hydrodynamic data accessible for perception by the lateral line system. In the case of the eyeless cavefish, the occurrence of double stagnation points resulted in the formation of two high‐pressure regions near the head, thereby enhancing their ability to perceive hydrodynamic pressure (Figure 2h). The concentrated and finely distributed lateral line system was adept at sensing the hydrodynamic stimuli within these high‐pressure regions. Thus, the aforementioned findings substantiated the Chinese eyeless cavefish's utilization of an EFPS, which is facilitated by the evolved unique head horn structure. This structure empowered the Chinese eyeless cavefish with an enhanced ability to explore their surroundings through flow field perception.

The impact of the head‐horn structure on the drag performance of eyeless cavefish was thoroughly examined. The comprehensive details can be found in Note S3 (Supporting Information). The eyeless cavefish, characterized by a distinctive head‐horn body shape, exhibited a higher drag coefficient compared to the streamlined body shape of the eyed cavefish (Figure S14, Supporting Information). However, the eyeless cavefish is a slow swimmer.^[^ ^36^, ^37^, ^38^ ^]^ The eyeless cavefish's sluggish swimming behavior is likely an adaptation to the challenging cave environments, providing a selective advantage for energy conservation in motion. Taking a comprehensive perspective, the eyeless cavefish's enhanced hydrodynamic perception capabilities outweigh the additional drag costs associated with locomotion. This compensation ultimately leads to an overall enhancement in the fitness of the troglobite within its dark, predator‐sparse habitat.

### 2.3 Enhanced Vortical Flow Field Perception

**Figure 3:**
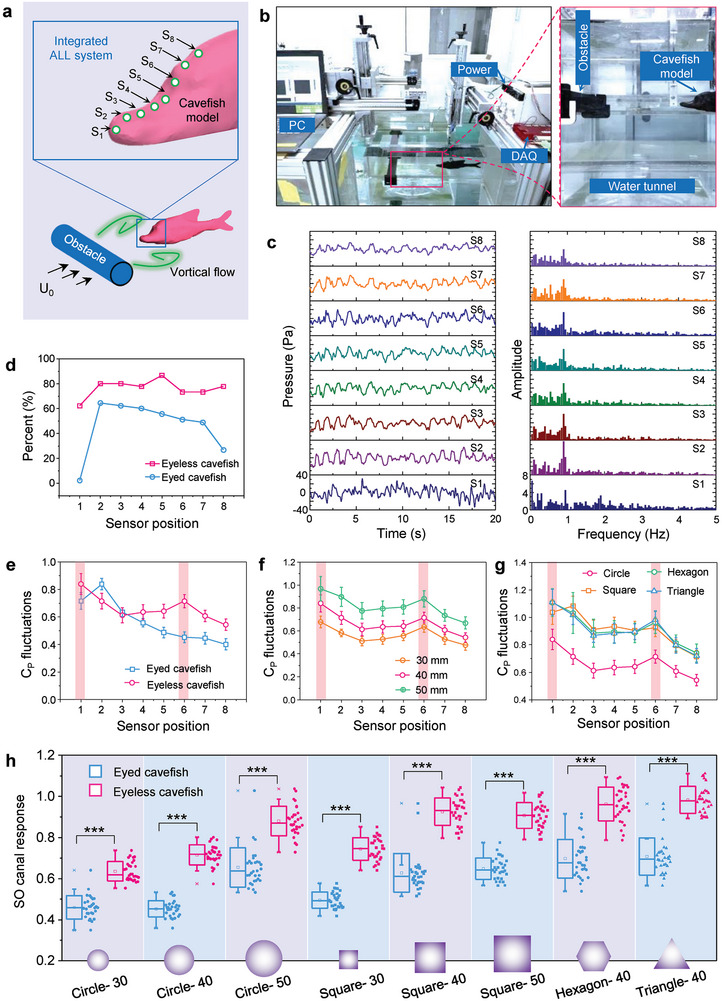
Enhanced hydrodynamic sensing in vortical flow field. a) Schematic illustration of the cavefish model for vortex streets detection. The inserted schematic on the right corner illustrates the pressure sampling positions of ALL system in cavefish model. b) Optical photographs present the experimental platform for vortical flow sensing by eyeless cavefish model. The cavefish model located behind obstacles horizontally placed in a flow tank. The signals of integrated ALL system were collected by a data acquisition (DAQ) board. c) Representative outputs of ALL system in eyeless cavefish model in time domain (left) and frequency domain (right), responding to an obstacle with a diameter of 40 mm. d) Dominant frequency recognition accuracy of ALL systems in cavefish models, responding to an obstacle with a diameter of 40 mm. (*n* = 45) e) Pressure fluctuations experienced by two cavefish models responding to a vortical field generated by a circular cylinder with a diameter of 40 mm. f) Pressure fluctuations experienced by eyeless cavefish models responding to circle obstacles with different sizes, namely, 30, 40, and 50 mm. g) Pressure fluctuations experienced by eyeless cavefish models responding to 40 mm obstacle with different sections, namely, circle, square, hexagon, and triangle. h) Maximum pressure fluctuations experienced by SO canal region within cavefish models responding to various obstacles; *n* = 30. Asterisks indicate statistically significant difference (^∗∗∗^ *P* < 0.001).

We designed and developed a cavefish model equipped with an ALL system capable of perceiving temporal hydrodynamic pressure (Figures S15 and S16, Supporting Information), inspired by EFPS employed by Chinese eyeless cavefish. The ALL system consisted of eight miniaturized pressure sensors, uniformly distributed around the model's head within the 0.2 BL region (**Figure** **3**a). Initially, we assessed the actual hydrodynamic pressure experienced by the cavefish model in uniform flow fields (Figures S17 and S18, Supporting Information). Under identical working conditions (angles of attack ranging from 0° to 15° and a flow velocity of 0.5 BL/s), the results demonstrated a close agreement between the hydrodynamic *C_P_* measured by the cavefish model and the *C_P_* response curve determined through CFD and PIV analysis (Figure 2e,f; Figure S9, Supporting Information). This not only validated the accuracy of the CFD and PIV techniques but also provided further confirmation of the utilization of EFPS by the Chinese eyeless cavefish.

In addition to sensing uniform flow fields, precise perception of vortical flow fields holds crucial significance in the daily activities of fish. Organized vortices generated by swimming fish or obstacles in flowing water offer valuable information concerning object size, distance, and shape.^[^ ^32^, ^39^ ^]^ The detection of vortices necessitates a functional lateral line system in fish.^[^ ^32^, ^40^ ^]^ To assess the vortical flow field perception capabilities of the eyed and eyeless cavefish, we subjected the cavefish models to Karman vortex streets produced by obstacles placed in a steady flow (Figure 3a,b). The ALL system within the cavefish model responded to the vortical flow field, exhibiting periodic outputs in the time domain and a dominant value in the frequency domain (Figure 3c; Figure S19, Supporting Information), thereby confirming the effectiveness of the experimental platform. The outputs of the ALL system displayed nonperiodic characteristics in the time domain and exhibited no prominent values in the frequency domain, when subjected to still water (Figure S20, Supporting Information) and stable flow conditions (Figure S21, Supporting Information). These findings further confirmed the reliability and robustness of the experimental platform.

To assess the improved hydrodynamic perception capability in eyeless cavefish, we employed two primary indicators, i.e., vortical flow frequency detection accuracy and response intensity. Frequency recognition accuracy served as a crucial criterion for evaluating vortex sensing performance. Remarkably, the ALL system within the eyeless cavefish model exhibited superior frequency recognition accuracy compared to that of the eyed cavefish model (Figure 3d; Figure S22, Supporting Information). This finding suggested that the distinctive body shape of the eyeless cavefish contributed to its enhanced perception of vortical fields.

The quantification of the ALL system response intensity was accomplished by measuring the standard deviation of pressure fluctuations caused by flow perturbations. Our findings revealed that in the eyed cavefish, the strongest fluctuations occurred at the front (S1 and S2), while they weakened downstream (Figure 3e; Figure S23, Supporting Information). In contrast, the eyeless cavefish exhibited two distinct peaks of fluctuation, positioned at the front (S1) and head horn (S6) regions (Figure 3e; Figure S23, Supporting Information). This phenomenon was consistently observed across various vortical fields generated by obstacles of different dimensions (Figure 3f) and sectional shapes (Figure 3g). Notably, as the size of the obstacle increased, the dimensions of the vortices expanded, resulting in higher outputs detected by the ALL system (Figure 3f). Additionally, when the obstacle shape transitioned from streamline (e.g., circle) to blunter (e.g., triangle), the flow perturbation intensified (Figure S24, Supporting Information), leading to elevated outputs in the ALL system (Figure 3g). Furthermore, we conducted a quantitative analysis of the enhanced hydrodynamic sensing capability of eyeless cavefish by examining the response of the SO canal. The results demonstrated that the lateral line canal system of the eyeless cavefish exhibited greater sensitivity compared to that of the eyed cavefish (*t*‐test; *P* < 0.001) (Figure 3h). Collectively, these findings provided validation for the EFPS facilitated by the unique head horn structure in the eyeless cavefish when encountering vortical flow fields.

**Figure 4:**
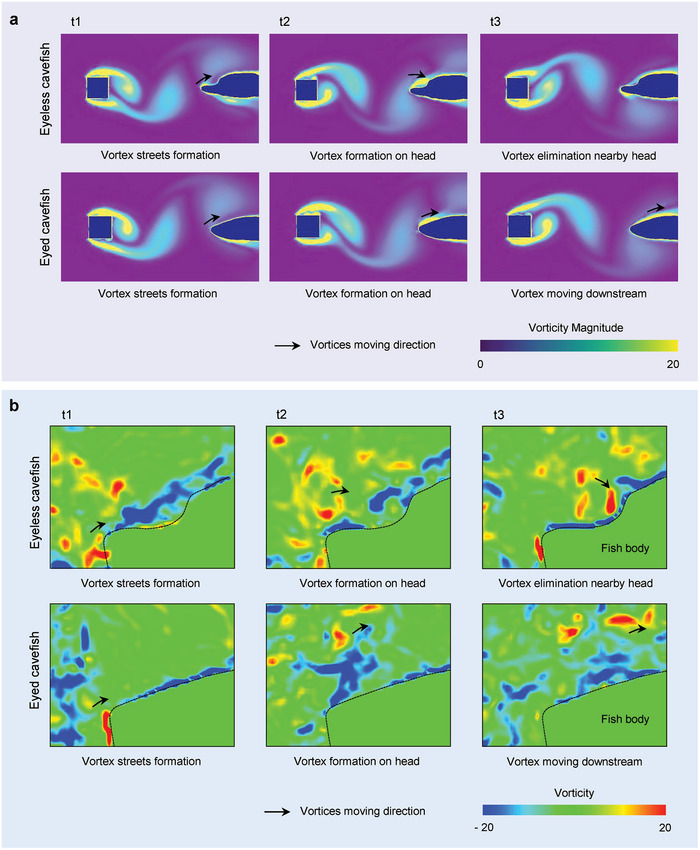
Enhanced hydrodynamic sensing mechanism in vortical flow field. a) Vortical flow field distribution around the cavefish head computed using CFD, illustrating enhanced perception mechanism in the vortical flow field. b) Vortical flow field distribution nearby cavefish head, measured by PIV. The black arrows indicate vortex. The black dash lines illustrate outlines of fish body.

To uncover the underlying mechanism, we conducted theoretical simulations to explore the distribution of the vortical flow field near the head of the cavefish. In the presence of obstacles positioned within a uniform flow, periodic vortices propagate downstream. When these vortical flows interacted with the cavefish model, vortex generation was observed at the snout. In the case of the eyeless cavefish with a head horn, this vortex initially moved along the body profile in a downstream direction and eventually dissipated in the head horn region (**Figure** **4**a; and Movie S1, Supporting Information). Conversely, in the eyed cavefish, the vortex moved toward the tail and became further detached from the fish body (Figure 4a; and Movie S2, Supporting Information). The simulation results were subsequently validated using PIV measurements conducted with a cavefish model (Figure 4b). The vortex, harboring valuable hydrodynamic information, converged within the head horn region, thereby substantially enhancing the hydrodynamic sensing capability of the eyeless cavefish.

### 2.4 Enhanced Underwater Obstacle Recognition

**Figure 5:**
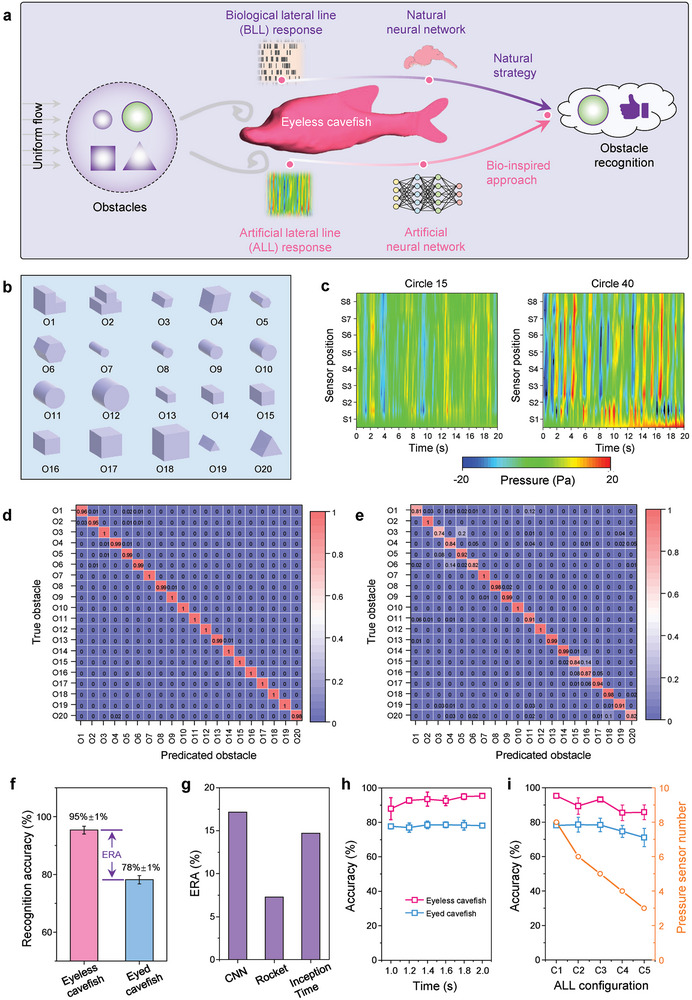
Obstacle recognition by cavefish models through machine learning. a) Schematic diagraph illustrates natural approach utilized by fish and bioinspired strategies employed by cavefish model for underwater obstacle recognition. b) Schematic illustration of obstacles used in the recognition through hydrodynamic perception. c) Examples of the corresponding hydrodynamic perception measurements decoded by the eyeless cavefish model for round obstacles with diameters of 15 (left) and 40 (right) mm, respectively. d,e), The confusion matrix of the eyeless and eyed cavefish models, respectively. f) Average classification accuracy of the cavefish models using CNN machine learning model. Error bars denote the standard derivation (SD) from 5 independent experiments. ERA, means the enhanced recognition accuracy achieved by eyeless cavefish, compared with eyed cavefish. g) ERA evaluated from three different machine learning models, including CNN, Rocket, and InceptionTime. h) Comparison of the recognition accuracy achievable with various stimulus times. Error bars denote the SD from 5 independent experiments. i) Comparison of the recognition accuracy achievable with various ALL configurations. The definition of ALL configurations was illustrated in Figure S31 (Supporting Information). C1: S1–S8; C2: S1–S6; C3: S1, S2, S4–S6; C4: S1, S2, S5, S6; C5: S1, S2, S6. Error bars denote the SD from 5 independent experiments.

The ultimate objective of hydrodynamic flow sensing in fish is to enable indispensable behaviors. For instance, fish can accurately identify different prey and objects solely based on vortical flow cues, as depicted in **Figure** **5**a. Beyond the perception of pure flow fields, it is equally important to validate the effectiveness of the EFPS at the behavioral level. With this in mind, we conceptually integrated machine learning algorithms into cavefish models to enable recognition of obstacles using pure hydrodynamic flow information (Figure 5a). We employed 20 obstacles with diverse shapes and dimensions (circle, square, triangle, “L,” ⊥, rhombus, and hexagon) (Figure 4b; Figure S25, Supporting Information) to replicate the fundamental elements found in complex cave environments, such as rocks, logs, and prey.

Distinct obstacles possessed varied surface profiles, resulting in diverse hydrodynamic stimuli (Figure S24, Supporting Information) that elicited distinct responses from the ALL systems, enabling effective classifications to be performed (Figure 5c). By aggregating the pulse outputs, we achieved “hydrodynamic imaging” through the integration of the ALL system into the cavefish model. For training purposes, a total of 7,200 images were randomly selected from the acquired signals, while the remaining 1,800 images constituted the test set. Leveraging hydrodynamic perception and a convolutional neural network (CNN)‐based machine learning model, we successfully demonstrated that the cavefish models were capable of accurately recognizing obstacles (Figure 5d–f). Notably, the eyeless cavefish model exhibited a remarkable recognition accuracy of 95%, surpassing that of the eyed cavefish model (Figure 5f). It is worth noting that the choice of the algorithm model greatly influences the recognition results. To identify the optimal model, we also tested two alternative machine learning algorithms, including Rocket and InceptionTime (Figures S26**–**S29, Supporting Information). The testing outcomes revealed that the CNN model achieved the highest accuracy among all the models evaluated (Figure S30, Supporting Information). The enhanced recognition accuracy, defined as the difference in accuracy between the two cavefish models, was notably highest for the CNN model, reaching a value of ≈17% (Figure 5g).

The evaluation of a sensory system relies on the crucial indicator of reaction time, where the most effective system achieves the highest accuracy using the shortest stimulus duration. Even when the stimulus period for the ALL system was reduced, the eyeless cavefish model maintained a higher recognition accuracy compared to the eyed cavefish model (Figure 5h). To achieve precise detection with minimal sensing elements, optimizing the arrangement of pressure‐sensor arrays is essential. Therefore, we modified the configuration of the ALL system, transitioning from all eight sensors (C1) to only three sensors (C5, two in the snout and one in the head horn region), to assess the performance of obstacle recognition (Figure S31, Supporting Information). The results demonstrated that the eyeless cavefish model exhibited ≈12% higher recognition accuracy than the eyed cavefish model (Figure 5i). These findings successfully validated the EFPS at the behavioral level of obstacle recognition and emphasized the significance of the optimal arrangement of ALL systems for reliable hydrodynamic learning and perception.

In addition to laboratory objects, the proposed EFPS enabled eyeless cavefish model to classify real‐world objects. To demonstrate this ability, we employed the eyeless cavefish model conceptually to classify clownfish toys based solely on hydrodynamic flow information (Figures S32–S34 and Note S4, Supporting Information). As expected, the eyeless cavefish model was able to classify real‐world clownfish toys, achieving a high accuracy of 100%. Furthermore, the proposed EFPS likely serves as an inspiration for engineers to strategically position flow sensors in underwater unmanned vehicles (UUVs) (Figure S35 and Note S5, Supporting Information).

## 3 Conclusion

Eyeless cavefish utilized hydrodynamics imaging, achieved by the mechanosensory lateral line system, to perform indispensable behaviors including swimming and obstacles recognition and to explore surrounding environments in further. In this study, we explored the functional significance of the head horn in *S. tianlinensis* for mediating lateral line sensing. CFD simulations, PIV, and ALL experiments showcased that the hydrodynamic stimuli impinging on the SO canal region in eyeless cavefish were much higher than those in eyed cavefish, both in uniform and vortical flow fields. This indicated that the unique head horn of eyeless cavefish most likely modifies flow fields and further enhances hydrodynamic perception, owing to the formation of double‐stagnation points in the uniform flow and the mechanism of double hydrodynamic impacts in a vortical flow field. As expected, the concentratedly distributed cephalic lateral line system evolved in the eyeless cavefish was able to precisely capture these enhanced hydrodynamic stimuli. In addition to flow field sensing level, the EFPS utilized by the eyeless cavefish was validated at behavior level in further. While experiencing hydrodynamic stimuli generated by different obstacles, the eyeless cavefish model can recognize up to 20 obstacles with much higher accuracy than the eyed cavefish model with the assistance of machine learning algorithms. The revealed EFPS enabled eyeless cavefish to achieve an excellent balance between the hydrodynamic perception capabilities and limited number of mechanoreceptors. This study demonstrated a promising application of the ALL system in bionic fish robots for flow analysis and object recognition.

## 4 Experimental Section

### Sample Preparation

In the previous investigation, the lateral line systems of *Sinocyclocheilus macrophthalmus* and *Sinocyclocheilus tianlinensis* were systematically examined by studying five individuals from each species.^[^ ^30^ ^]^ Additionally, the body shapes of these two cavefish species were validated: *S. macrophthalmus* possesses a fusiform body shape, while the eyeless *S. tianlinensis* showcases a distinctive head horn body shape, consistent with previous studies.^[^ ^26^, ^31^ ^]^ In addition, the morphological variation within species was neglected in this study. For the sake of convenience, *S. tianlinensis* (collected from karst caves in Tianlin County, Guangxi Zhuang Autonomous Region, China) with a BL of 8 cm and *S. macrophthalmus* (collected from karst caves in Du'an County, Guangxi Zhuang Autonomous Region, China) with a BL of 7.6 cm were used as prototypes to create high‐fidelity 3D models. The animals were collected in 2018, and all biological experiments were accomplished before 2020. Fish cultivation and experimentation were performed in accordance with the regulations on the treatment of experimental animals of Beijing, China.

### 3D Digital Model Creation

Living specimens were euthanized using an overdose of MS‐222. After fixation in formalin solution for ≈6 months, the specimens were washed in running water for ≈24 h to remove the remaining chemicals. The surfaces of the fish were coated with a thin layer of developer (DPT‐5, Shanghai Xinmeida Flaw Detection Material Co., Ltd., China) to reduce reflections. A laser scanner (ZGScan 313; Wuhan Zhongguan Automation Technology Co., Ltd., China) with a spatial resolution of 30 µm was used to obtain a virtual 3D model of the fish surface. Finally, the 3D digital models of the two cavefish species were created using reverse engineering. For convenience, the fish body was assumed as rigid, neglecting the skin's soft and deformations. The final digital models of the cavefish were amplified thrice to facilitate the development of cavefish models with integrated ALL systems. The amplified models were utilized in all subsequent experiments.

### Computational Fluid Dynamics: Cavefish in Uniform Flow

CFD simulations were performed using the commercial software Workbench 17.2 (Ansys) running on a workstation (Intel (R) Xeon (R) CPU E5‐2630 v4 @2.2 GHz, 32GB RAM, NVIDIA Quadro M4000, Win10, 64 bit). The 3D models of the cavefish were placed in the center of the calculation domain, which was a cube with a side length of 10 BL (Figure S4a, Supporting Information). The leading and trailing surfaces of the bodies were defined as the pressure inlets and outlets, respectively. The flow velocity at the inlet is 0.5 BL/s, i.e., 0.12 and 0.11 m s^−1^ for *S. tianlinensis* and *S. macrophthalmus*, respectively. For convenience, hydrodynamic stimuli acting on the midline of the cavefish model were extracted (Figure S4b, Supporting Information). Self‐consistency was tested using CFD cases with coarse, fine, and fine grids (Figures S4c,d and S5, Supporting Information). A grid of 150 µm was selected considering the time cost and calculation efficiency.

### Vortical Fields Behind Different Shape Obstacles

A 2D model was created in this study for better efficiency. Obstacles, namely, circles, squares, triangles, and hexagons, with equivalent diameters of 40 mm were placed in a rectangular calculation region of 70 × 35 cm^2^. The k‐ω SST turbulence model was employed for a Reynolds number Re ≈ 4000. A no‐slip boundary layer condition was employed for the cylindrical surface. The left and right‐hand side edges of the rectangular region were set as the velocity inlet at 0.1 m s^−1^ for convenience and pressure outlet of 0 Pa, respectively. For quantitative analysis, the hydrodynamic pressures at points 21, 22, and 23 cm downstream of the obstacle were extracted and analyzed.

### Cavefish in Vortical Flow

The 2D cavefish model was placed at the center of the calculation domain, a square with a side length of 10 BL. Obstacle, a square with a side length of 40 mm, was placed in front of the cavefish model. The interval between the obstacle center and the cavefish snout was set at 20 cm. Flow velocity at the inlet and pressure at the outlet were set as 0.17 m s^−1^ and 0 Pa, respectively. Finally, the flow velocity field near the head of the cavefish was studied.

### Particle Image Velocimetry: Cavefish in Uniform Flow

Using 3D printing techniques, physical models of cavefish were obtained and coated with a thin layer of black paint to reduce optical reflection. The physical models of the cavefish were placed in the center of a flow tank (70 × 35 × 40 cm^3^) (Figure S7, Supporting Information). The inlet flow velocity was set as ≈0.13 m s^−1^. The flow tank was seeded with floating glass beads (diameter 10–20 µm). Seeding particles were illuminated using a laser sheet with a thickness of ≈1 mm generated using a 10 W dual‐cavity pulsed laser (SM‐SEMI‐10W, ND: YLF, 527 nm, Beijing MicroVec Inc., China). Images were captured above the cavefish head using a high‐speed camera (Fastcam Mini WX100, 500 frames per second, Photron, Japan) fitted with a 100 mm macro lens (Canon, Japan) whose axis was normal to the light sheet. The PIV calculations were performed using MicroVec v3.6.2 (Beijing MicroVec Inc.) with 60 sequential images.

### Cavefish in Vortical Flow

The inlet flow velocity was set as ≈0.17 m s^−1^. Images were captured above the cavefish head using a high‐speed camera (Fastcam Mini WX100, 250 frames per second, Photron, Japan) fitted with a 100 mm macro lens (Conon, Japan) whose axis was normal to the light sheet. Particle image velocimetry (PIV) calculations were performed using MicroVec v3.6.2 (Beijing MicroVec Inc.) with two images in sequence.

### Development of Bioinspired Cavefish Model

Cavefish models have passive rigid tails and pressure‐tapped heads and were manufactured using rapid prototyping and lacquer coating. Inside each head section, eight holes were connected to the pressure sensors using silicon tubes. Holes with an internal diameter of 2 mm were distributed around the model heads within the 0.2 BL region. The spatial intervals between adjacent holes along the model profiles were 8 mm (*Sinocyclocheilus tianlinensis* model) and 7.5 mm (*S. macrophthalmus* model). The ALL system was composed of eight commercial pressure sensors (MS5401‐AM, Measurement SpecialtiesTM). The output of the pressure sensors was amplified ≈500 times, with a custom‐designed amplification circuit (Figure S16, Supporting Information). The pressure sensors were sealed in a watertight box that housed the electronic circuits necessary for the data. The sensitivity of the ALL system is listed in Table S1 (Supporting Information).

### Cavefish Model Hydrodynamic Perception: Uniform Flow

The bioinspired cavefish model equipped with an ALL system was placed at the center of the flow tank, ≈12 cm below the water surface, to reduce the influence of surface waves (Figure S17, Supporting Information). The AOA, defined as the angle between the incoming flow and the longitudinal body axis of the fish model, was varied from 0° to 15° in steps of 5°. Incoming flow parallel to the longitudinal body axis of the cavefish model: 0°. Before conducting the experiment, the laminarity and velocity of the flow were verified using PIV at different rotational speeds of the flow pump. In these experiments, the flow velocity was ≈0.13 m s^−1^. Three trials were conducted for each angle of attack. All pressure sensors were powered using a DC power source (PMM35, KIKUSUI, output ± 2.5 V). The voltage outputs from ALL were parallelly and real‐time collected by a data acquisition (DAQ) board (T7Pro, 16 bit, LabJack, sampling rate of 500 Hz) and low‐pass filtered (10 Hz). The detailed experimental procedures are available in Note S1 (Supporting Information).

### Vortical Flow

The cavefish model was located at the center of a flow tank filled with water at a depth of 30 cm, ≈15 cm below the water surface, to reduce the influence of surface waves. The obstacles were placed horizontally in a flow tank in front of a cavefish model. The interval between the obstacle center and the cavefish snout was maintained at 20 cm. The incoming flow velocity was set to 0.17 m s^−1^. A DAQ board was employed to collect the voltage outputs from the ALL in a parallel and real‐time manner, with a sampling frequency of 230 Hz and a low‐pass filter of 10 Hz.

### Machine Learning‐Enabled Obstacle Recognition

To demonstrate the hydrodynamic stimulus recognition, the obstacles comprised a circle, square, triangle, L, ⊥, rhombus, and hexagon. To simulate the “touch‐in‐distance” capability in biological lateral line, the obstacle was placed in front of the cavefish model instrumented with ALL system. In each trial, the incoming flow velocity was ≈0.17 m s^−1^. Forty‐five trials (each trial was maintained for 90 s) were conducted for each obstacle with a 2 s interval between trials, and the data from the ALL system were recorded at 230 Hz using a DAQ system. With the hydrodynamic touch datasets, the responses of the ALL system were aggregated for each trial to form the “hydrodynamic image” for obstacle recognition. Each “hydrodynamic image” was an 8 × 1 matrix, where each element corresponded to the aggregated responses from a single sensor element.

For the dataset containing 20 classes of hydrodynamic images generated by different obstacles, a ratio of 80–20% was randomly selected and divided into training and testing sets for each class. Subsequently, 80% of the data were used to train the proposed model, whereas the remaining 20% was utilized to further test the optimal parameters after calibration.

To determine an optimized model, obstacle recognition was performed using three different machine learning methods: CNN, rocket, and InceptionTime models. Each experiment was repeated five times using the same parameters for the training and testing datasets, and the results were compiled from the five training instances. Similarly, the average classification accuracy for the five training instances was reported. The details of the machine learning process were as follows:

1. *CNN model*: The CNN model architecture included two convolution layers with a kernel size of 9. Each convolutional layer was followed by an activation function of the sigmoid and average pooling layers with a size of 11. Next, the output of the final average pooling was flattened to add a dense layer in which the dimensionality of the output was 20, that was, the number of classes. The output probability of each class was calculated using a softmax function. The CNN was trained to minimize the categorical cross‐entropy loss. The Adam optimization algorithm was implemented with an initial learning rate of 0.001 to train the CNN for 500 epochs with a batch size of 128.
2. *Rocket model*: The Rocket machine‐learning network transformed a series of hydrodynamic imaging data into features using 200 random convolutional kernels, with a maximum number of dilations per kernel of 32. Each feature applied a proportion of positive value (PPV) pooling to produce PPV values that must concatenate before training the ridge regression classifier. The classifier wrapped the rocket transformer using the Ridge ClassifierCV.
3. *InceptionTime model*: The InceptionTime uses multiple filters of different sizes to analyze variant time series features simultaneously. Two depths of inception module and 16 filters of 1‐dimension convolution were employed. The number of bottleneck 1‐dimension convolution filter was 8. The InceptionTime added max pooling in between inception modules. This was followed by global average pooling, dense layers, and a softmax function for prediction after raw hydrodynamic data passed through inception modules. The model was trained to minimize the categorical cross‐entropy loss. The Adam optimization algorithm was implemented with an initial learning rate of 0.0001 to train 50 epochs with a batch size of 32.

### Statistical Analysis

Statistical analysis was conducted through a two‐tailed Student's t‐test. The significant differences were showcased by *** < 0.001. The data were presented as mean ± standard deviation. At least three independent trials were performed in triplicates for each experiment.

## Conflict of Interest

The authors declare no conflict of interest.

## Author Contributions

Z.M. And Z.G. contributed equally to this work. Z.M. and Y.J. conceived the project and designed the experiments. Z.M., Z.G., and Y.J. performed the experiments and analyzed the experimental data. Z.M., Y.J., P.W., H.C., and Z.D. performed the computational fluid dynamics simulations. Z.M., Z.G., Y.J., P.W., Y.Z., and H.C. proposed an enhancement mechanism for hydrodynamic perception induced by a head horn body shape. Z.M., Y.J., and Y.Z. performed the particle image velocimetry experiments. Z.M., Z.G., and Y.J. designed and fabricated the bioinspired cavefish models and performed hydrodynamic experiments. Z.M. and C.Y. conducted machine learning for obstacle recognition. Z.M., Y.J., and D.Z. wrote the manuscript.

## Supporting information



Supporting Information

Supplemental Movie 1

Supplemental Movie 2

## Data Availability

The data that support the findings of this study are available from the corresponding author upon reasonable request.
