# Supplementary material for: Head Horn Enhances Hydrodynamic Perception in Eyeless Cavefish
Source: Adv Sci (Weinh). 2024 Sep 23;11(44):2406707. doi: 10.1002/advs.202406707 (PMC11600165; doi:10.1002/advs.202406707)
Supplement: Supplementary file 1 — Supporting Information [file ADVS-11-2406707-s001.docx]

Supporting Information for

Head horn enhances hydrodynamic perception in eyeless cavefish

Zhiqiang Ma, Zheng Gong, Yonggang Jiang,*, Peng Wu, Changxin You, Zihao Dong, Hongchao Cao, Zhen Yang, Yahui Zhao, Huawei Chen, Deyuan Zhang

Zhiqiang Ma, Zheng Gong, Yonggang Jiang, Zihao Dong, Hongchao Cao, Zhen Yang, Huawei Chen, Deyuan Zhang

Institute of Bionic and Micro-Nano Systems, School of Mechanical Engineering and Automation, Beihang University, Beijing 100191, China

E-mail: [jiangyg@buaa.edu.cn](mailto:jiangyg@buaa.edu.cn)

Yonggang Jiang

International Research Institute for Multidisciplinary Science, Beihang University, Beijing 100191, China

Peng Wu

Artificial Organ Technology Lab, Bio-manufacturing Research Center, School of Mechanical and Electric Engineering, Soochow University, Suzhou 215021, China

Changxin You

Centre for Artificial Intelligence and Robotics, Hong Kong Institute of Science & Innovation, Chinese Academy of Sciences, Hong Kong, 999077, China

Yahui Zhao

Key Laboratory of Zoological Systematics and Evolution, Institute of Zoology, Chinese Academy of Sciences, Beijing, 100101, China

Supplementary Notes

Note S1: Cavefish model experiment in uniform flow

A bioinspired cavefish model was placed in the center of a flow tank (water depth: 30 cm) (**Fig. S16**). To reduce the influence of surface waves, cavefish models (length: ~ 24 cm) were positioned 12 cm below the water surface. Before conducting an experiment, the laminarity and velocity of the flow was checked by particle image velocimetry (PIV) at different flow speeds (rotational speeds of the flow pump). In the experiments, rotational speed was fixed at 680 revolutions per minute (RPM) and the corresponding flow velocity was about 0.13 m/s. Angles of attack (AOA) were defined as the angle between incoming flow and the longitudinal body axis of the cavefish model. AOA varied from 0° to 15° with a step of 5°. 0° refers to incoming flow parallel to the longitudinal body axis of the cavefish model. Three trials were performed at each angle of attack. Pressure sensors of the artificial lateral line system (ALL) were powered by a DC power source (PMM35, KIKUSUI, output ± 2.5 V). Voltage outputs from the ALL were fed into a data acquisition board (T7Pro, 16 bit, LabJack, sampling rate 500 Hz) and low-pass (10 Hz) filtered.

Before each experiment, pressure sensors were calibrated by submerging the ALL systems at different depths and recording the corresponding output voltage signals. The voltage outputs of the pressure sensors exhibited a linear relationship with the pressure variation, as shown in **Fig. S17a**. The sensitivity (SE) of the pressure sensors is listed in **Table S1**.

Experimental conditions were still water, running water and again still water (**Fig. S17b**). Each test period lasted for about 120s. In the first 20s still water period, mean values of pressure are denoted V_1_. In the second still water period, mean values of pressure were recorded at the end of the period. Pressure values are denoted as V_2_. In the running water period, mean values of pressure, recorded in the middle of a 10 s period, was denoted V_3_. Sometimes pressure values of a sensor were instable. To account for this, stability of the pressure sensors was calculated according to:

| $\varepsilon=\frac{V_{2}-V_{1}}{V_{1}}$ | (1) |
| --- | --- |

with *ε* = relative error. If *ε* was larger than 5%, i.e. if a sensor had a DC drift, the recording was repeated. The pressure output of each sensor was calculated according to the formula:

| $S_{\text{i}}=\frac{V_{3}-V_{1}}{SE_{i}}$ | (2) |
| --- | --- |

with *i* = number of the respective pressure sensor, *SE* is the sensitivity of pressure sensors. The hydrodynamic pressure response (si) of the ALL was acquired by:

| $\text{si}=S_{i}+S_{9}$ | (3) |
| --- | --- |

where S_9_ is the pressure output of sensor 9. Pressure distributions along the cavefish heads were obtained as shown in **Fig. S17c**.

Note S2: Enhanced flow velocity perception in eyeless cavefish

The hydrodynamic stimulus that drives SNs is flow velocity [R1]. However, local flow velocity cannot be simply defined in CFD simulations due to the existence of a boundary layer. Previous studies adopt wall shear stress *τ_w_* as the hydrodynamic stimulus of SNs [R2-R5]. The skin friction coefficient (*C_f_*), a normalized version of shear stress, can be calculated as:

| $C_{\text{f}}=\frac{\tau_{W}}{0.5\rho U_{0}^{2}}$ | (4) |
| --- | --- |

The eyeless cavefish possesses two information rich areas of C_f_ (**Fig. S12**): one is located at the snout, and the other at the head-horn region. Quantitative analysis shows that C_f_ peaks both at the snout and at the head-horn (**Fig. S13a**). The SNs in the snout region (**Fig. 1b**) are stimulated by the first peak shear stress. The second peak shear stress can be perceived by the SNs located close to the head-horn. In the eyed cavefish, the maximum shear stress appears only in the snout region (**Fig. S12** and **Fig. S13b**).

The stimulus C_f_ that impinges on the SNs within the SO region was analyzed. In the eyeless cavefish, the maximum C_f_ within the SO region is significantly higher than that in the SO’s symmetrical region at the ventral side with a fusiform profile (**Fig. S13c**). In contrast, in the eyed cavefish, C_f_ within the SO region is comparable to that within its symmetrical region at the ventral side, which is much smaller than C_f_ within the SO region of the eyeless cavefish. According to our calculations, the flow stimulus experienced by SNs in the SO region of the eyeless cavefish is stronger than that of the eyed cavefish. This holds true for different angles of attack and different flow velocities (**Figs. S13c, S13d**). The unique head-horn in the eyeless cavefish obviously facilitates hydrodynamic perception by enhancing the hydrodynamic stimuli that impinge on the SNs of the lateral line system.

Note S3: Drag coefficient evaluation

The drag coefficient (*C_d_*), a normalized version of drag force, can be calculated as:

| $C_{\text{d}}=\frac{F}{0.5\rho AU_{0}^{2}}$ | (5) |
| --- | --- |

where, *F* is the drag force determined by the CFD simulation, *U_0_* is incoming flow velocity, *ρ* is water density, and *A* is the surface area of the fish’s body. As expected, the eyeless cavefish with a unique head-horn body shape exhibited higher drag coefficient than the eyed cavefish with a streamlined body shape (**Fig. S14**). Previous studies have indicated that the eyeless cavefish exhibits a slow swimming behavior [R6-R8]. This sluggish swimming behavior in the eyeless cavefish was likely an adaptation to the oligotrophic subterranean environments, characterized by low predation risk and limited nutrient content. Consequently, there exists a selective advantage for energy conservation in movement.

This study validated that unique head-horn structures rendered the eyeless cavefish with enhanced hydrodynamic sensing performance and obstacle recognition capability compared to eyed cavefish, using multiple methods (CFD, PIV, cavefish model integrated with artificial lateral line systems).

From a comprehensive perspective, the enhanced hydrodynamic perception capabilities in the eyeless cavefish surpassed the additional drag costs linked to locomotion. As a result, this compensation leads to an overall fitness improvement of the troglobite within its dark, predator-scarce habitat.

Note S4: Clownfish toys recognition by eyeless cavefish model

In addition to laboratory objects, the eyeless cavefish model demonstrated precise classification of real-world objects. To showcase this ability, we employed the eyeless cavefish model conceptually to classify clownfish toys based solely on hydrodynamic flow information. These two clownfish toys shared similar shapes but differed in dimensions—referred to as the large clownfish (10 cm in length) and the small clownfish (8 cm in length). Placed in front of the eyeless cavefish model, the clownfish toys were positioned approximately 20 cm apart. The incoming flow velocity was standardized at 0.17 m/s. Real-time data from the ALL system within the eyeless cavefish model was captured by the DAQ system, with a sampling frequency of 230 Hz and a low-pass filter set at 10 Hz. The signal outputs from the eyeless cavefish exhibited periodic patterns in the time domain and a prominent peak in the frequency domain (**Fig. S32**, **S33**). This observation confirmed the eyeless cavefish's successful capture of the vortical flow fields generated by the clownfish toys.

Leveraging the discerned hydrodynamic data, a CNN model was employed to classify the clownfish toys. 80% of the dataset was allocated for training the CNN model, with the remaining 20% reserved for fine-tuning and parameter testing. As anticipated, the outcomes substantiated that the eyeless cavefish model could accurately classify these clownfish toys with an impressive 100% accuracy rate (**Fig. S34**). Notably, even when transitioning from the ALL system to the C1 configuration (comprising only three pressure sensors, as illustrated in **Fig. S34**), the classification accuracy remained notably high at 100%.

Note S5: Demonstration of EFPS application in UUVs

The approach observed in eyeless cavefish offers valuable insights for engineers aiming to optimize the spatial configuration of flow sensors in underwater unmanned vehicles (UUVs). This methodology involves three key stages: the creation of a 3D model of the UUV, the analysis of surrounding flow fields, and the strategic arrangement of flow sensors. To elucidate this process, we executed a demonstration of the EFPS's practical application in a miniature UUV (**Fig. S35a**). Considering computational efficiency, we developed a simplified 3D model while preserving essential geometric characteristics, as depicted in **Fig. S35b**. The final dimensions of the UUV model were 200 mm (length) × 200 mm (width) × 155 mm (height).

Subsequently, an analysis of the flow fields surrounding the UUV was conducted. Computational Fluid Dynamics (CFD) simulations were carried out using the commercial software Multiphysics 5.4. The 3D model of the UUV was positioned at the center of the computational domain, represented by a cuboid with dimensions of 10 BL (length) × 8 BL (width) × 8 BL (height). The leading and trailing surfaces of the UUV were designated as pressure inlets and outlets, respectively. At the inlet, a flow velocity of 0.5 BL/s (equivalent to 0.06 m/s) was prescribed, while the outlet was set to 0 Pa. The *k*-*ε* SPF turbulence model was employed for a Reynolds number of approximately 12000. A no-slip boundary layer condition was imposed on the surface of the UUV. Lastly, the distribution of the flow pressure field on the UUV surface was thoroughly examined.

As anticipated, the simulation outcomes demonstrated three prominent high-pressure zones around the UUV, as showcased in **Fig. S35c**. Drawing inspiration from the EFPS mechanism observed in eyeless cavefish, we propose the placement of three standard pressure sensors at these identified locations to capture comprehensive hemodynamic data, as depicted in **Fig. S35d**.

Supplementary Figures

**Fig. S1. Complicated cave environments where cavefish survive.**

**Fig. S2. Body shape in some troglobiotic cavefish *Sinocyclocheilus*.** Head-horn body shape is probably the result of convergent evolution. Images of *S. rhinocerous*, *S. broadihornes*, *S. hyalinus*, *S. aquihornes*, *S. anatirostris*, *S.* *anophthalmus* and *S. furcodorsalis* are modified from Romero et al. 2008 [R9]. Image of *S. jinxiensis* is adapted from Zheng et al. 2013 [R10].

**Fig. S3. Distribution of the cephalic canal pores in *Sinocyclocheilus macrophthalmus* (left) and *S. tianlinensis* (right).** (a) Lateral view. (b) Dorsal view. (c) Ventral view. Blue and red circles represent canal pores. IO, infraorbital canal; MD, mandibular canal; OT, otic canal; PO, postotic canal; PRO, preopercular canal; SO, supraorbital canal; ST, supratemporal canal; T, temporal canal. (reproduced from Ma et al. [R11])


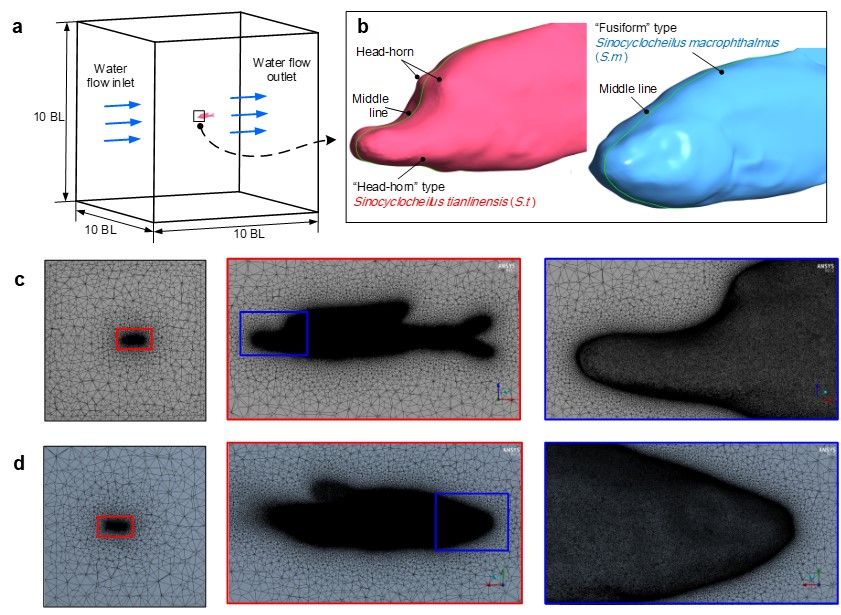


**Fig. S4. CFD simulation configuration and mesh generation.** (a) Configuration used for the 3D simulations. Calculation domain was a cube with a side length 10 BL (body length). Cavefish models were placed in the center of the calculation domain. (b) 3D digital models of the eyeless cavefish *Sinocyclocheilus tianlinensis* (left) and the eyed cavefish *S. macrophthalmus* (right). Mesh width in case of the eyeless cavefish model *S. tianlinensis* (c) and the eyed cavefish model *S. macrophthalmus* (d).

**Fig. S5. CFD simulation mesh optimization**. Grid independent analysis of the eyeless cavefish model *Sinocyclocheilus tianlinensis* (a) and the eyed cavefish model *S. macrophthalmus* (b).

**Fig. S6. Pressure coefficient (C_P_) distribution on fish bodies.** AOA 5°, 10° and 15°. Model of the eyeless cavefish *Sinocyclocheilus tianlinensis*. (a) and the eyed cavefish *S. macrophthalmus* (b).

**Fig. S7. Experimental platform for PIV measurements.** Scheme (a) and photograph (b) of the experimental setup. (c) and (d): Photographs (exposure time 1/500 s) reveal path lines of particles illuminated by the laser sheet (c, eyeless cavefish model; d eyed cavefish model). The artificial particle streak images made by overlaying ten consecutive video frames. Pink lines illustrate the edges of the flow domain close to the cavefish based on PIV calculations.

**Fig. S8. Validation of CFD results with PIV results, for flow field distribution nearby cavefish head.** AOAs varied between 5° and 15°. Incoming flow velocity was 0.5 BL/s. White arrows: stagnation points at the snout region of the cavefish models. Pink arrows: stagnation point at the head horn in the eyeless cavefish model. Note that there is no second stagnation point in the flow field around the eyed cavefish model.

**Fig. S9. Distribution of pressure coefficient *C_P_* as function of surface location.** Calculations were done for the head of the eyeless cavefish *Sinocyclocheilus tianlinensis* (top) and the eyed cavefish *S. macrophthalmus* (bottom). Flow velocity was 0.13 m/s, AOA was 5°, 10° and 15°. CFD, computational fluid dynamics; PIV, particle image velocimetry; ALL, artificial lateral line. SO, supraorbital canal.

**Fig. S10. Distribution of hydrodynamic pressure gradients along head region.** Lateral line canal neuromasts respond in proportion to pressure gradients along lateral line canals. To account for this, pressure coefficient gradients were calculated. (a, b): Pressure gradient coefficients (ΨC_P_), distributed along the midline of the eyeless and the eyed cavefish, respectively. Flow velocity was 0.5 BL/s, AOAs (angles of attack) were 0°, 5°, 10° and 15°. SO, supraorbital canal. (c, d): Comparison of lateral line stimuli that imping on the SO canal located at the ventral side in the species investigated. (c) AOAs varied between 0° and 15° (flow velocity 0.5 BL/s. (d) Flow velocities varied between 0.25 and 1.0 BL/s (AOA = 0°).

**Fig. S11. Distribution of hydrodynamic pressure differences along fish head.** Pressure coefficient difference (ΔC_P_) distribution along the midline of blind cavefish (a) and eyed cavefish (b). Incoming flow velocity was 0.5 BL/s, AOAs (angles of attack) were 0°, 5°, 10° and 15°. SO, supraorbital canal. c, d: Comparison of lateral line stimuli that impinged on the SO canal and its corresponding location at the ventral side of the cavefishes. In (c) AOAs varied between 0° and 15° (incoming flow velocity U_0_ was 0.5 BL/s), in (d) flow velocity varied between 0.25 and 1.0 BL/s AOA = 0°.

**Fig. S12. Relationship between cephalic superficial lateral line system and skin friction coefficient (C_f_) distribution on fish body at varied AOAs.**

**Fig. S13. Enhanced flow velocity perception induced by head horn structure.** a and b: The C_f_ distribution along the middle line in the eyeless cavefish (left) and the eyed cavefish right). Incoming uniform flow velocity is 0.5 BL/s, AOAs (angles of attack) varied between 0° and 15°. c and d: Comparison of the flow that impinges on the SO canal and its corresponding location at the ventral side in the two cavefish species for different AOAs (U_0_ = 0.5 BL/s) (c), and flow velocities (AOA = 0°) (d).

**Fig. S14. Drag coefficient evaluated for the cavefish.**

**Fig. S15. Design and development of the bioinspired cavefish model for hydrodynamic pressure sensing.** (a) Structural configuration for cavefish model integrated with an artificial lateral line system, which is composed of 8 pressure sensors. (b) Optical image of the developed eyeless cavefish model.

**Fig. S16. Design and development of an artificial lateral line (ALL) system.** (a) Amplification circuit of MS5401-AM pressure sensors. (b) Printed circuit board (PCB) design for MS5401-AM pressure sensor array, showing front side (left) and back side (right). (c) Fabricated MS5401-AM pressure sensor array with electronic elements, showing front side (left) and back side (right).

**Fig. S17. Uniform flow experimental platform for cavefish models.** (a) Schematic illustration. (b) Optical photograph.

**Fig. S18. Representative analysis process of the eyeless cavefish model in uniform flow fields.** The flow velocity and AOA were maintained as 0.13 m/s (~ 0.5 BL/s) and 0°, respectively. (a) Calibration of the ALL system. S = pressure sensor. (b) Voltage outputs of the ALL system in the time domain. Flow velocity was 0 m/s (first blue area), 0.13 m/s (pink area) and again 0 m/s (second blue area). White areas: flow velocity was increased from 0 m/s to 0.13 m/s (left) and then, after a steady state period, back to 0 m/s (right). (c) Pressure outputs of sensors 1 to 8 of the ALL system. Flow velocity and AOA were 0.13 m/s (~ 0.5 BL/s) and 0°, respectively.

**Fig. S19. Representative outputs of ALL system in eyed cavefish model, responding to an obstacle with a diameter of 40 mm.** Left: time domain; right: frequency domain.

**Fig. S20. Representative outputs of ALL system in eyeless cavefish model located in still water.** Left: time domain; right: frequency domain. The time domain results were almost stale. It was clear that there was no dominate peaks in the frequency domain.

**Fig. S21. Representative outputs of ALL system in eyeless cavefish model, responding to a stable incoming flow.** Left: time domain; right: frequency domain. The time domain results were turbulent, without specific periodicity. And it was clear that there was no dominate peaks in the frequency domain.

**Fig. S22. Frequency distribution of ALL systems in cavefish models, responding to an obstacle with a diameter of 40 mm.** left: eyeless cavefish model; right: eyed cavefish model. n = 45.

**Fig. S23. Enhanced hydrodynamic perception capability in various vortical flow fields generated behind different obstacles.**

**Fig. S24. Simulation for the pressure distribution within vortex streets generated by different shape obstacles with an equivalent diameter of 40 mm.** The incoming flow velocity is maintained at 0.1 m/s.

**Fig. S25. Photographs of obstacles used for recognition experiments.**

**Fig. S26. Obstacle recognition through Rocket model.** The confusion matrix of the eyeless (a) and eyed (b) cavefish models, respectively.

**Fig. S27. Obstacle recognition accuracy through Rocket model.** Error bars denote the SD (standard deviation) from 5 independent experiments.

**Fig. S28. Obstacle recognition through InceptionTime model.** The confusion matrix of the eyeless (a) and eyed (b) cavefish models, respectively.

**Fig. S29. Obstacle identification accuracy through InceptionTime model.** Error bars denote the SD (standard deviation) from 5 independent experiments.

**Fig. S30. Obstacle recognition accuracy comparison among three machine learning models.**

**Fig. S31. Artificial lateral line configuration definition.** Green circles present the activated pressure sensor employed in ALL system.

**Fig. S32. Representative results from eyeless cavefish model detecting large clownfish toy.** Left: time domain; right: frequency domain.

**Fig. S33. Representative results from eyeless cavefish model detecting small clownfish toy.** Left: time domain; right: frequency domain.

**Fig. S34. Clownfish toys recognition through eyeless cavefish model powered by CNN model.** The confusion matrix of the eyeless cavefish model integrated with (a) C1 and (b) C5 types ALL systems. (c) Comparison of the recognition accuracy achievable with different artificial lateral line (ALL) configurations.

**Fig. S35. Application of EFPS for underwater unmanned vehicles (UUVs).** (a) Image of UUV. (b) Created simplified 3D model of UUV. (c) Flow field distribution nearby UUV. (d) Potential flow sensor arrangement on the UUV.

Supplementary Tables

**Table S1. The sensitivities of pressure sensors within ALL system employed in cavefish models.**

| Pressure sensor number | Sensitivity (mV/Pa) |
| --- | --- |
| S1 | 1.22 |
| S2 | 1.16 |
| S3 | 1.18 |
| S4 | 1.18 |
| S5 | 1.2 |
| S6 | 1.18 |
| S7 | 1.19 |
| S8 | 1.16 |
| S9 | 1.2 |

Supplementary Video

**Movie S1. Vortical flow field distribution nearby eyeless cavefish experienced in vortical flow fields.**

**Movie S2.** **Vortical flow field distribution nearby eyed cavefish experienced in vortical flow fields.**

Supplementary References

[R1] A. B. A. Kroese, N. A. M. Schellart, *J. Neurophysiol.* **1992**, 68, 2212-2221.

[R2] S. P. Windsor, S. E. Norris, S. M. Cameron, G. D. Mallinson, J. C. Montgomery, *J. Exp. Biol.* **2010**, 213, 3819-3831.

[R3] S. P. Windsor, S. E. Norris, S. M. Cameron, G. D. Mallinson, J. C. Montgomery, *J. Exp. Biol.* **2010**, 213, 3832-3842.

[R4] M. A. Rapo, H. Jiang, M. Grosenbaugh, S. Coombs, *J. Exp. Biol.* **2009**, 212, 1494-1505.

[R5] S. P. Windsor, M. J. McHenry, *Integr. Comp. Biol.* **2009**, 49, 691-701.

[R6] M. L. Niemiller, T.L. Poulson, Subterranean fishes of North America: Amblyopsidae. In: Trajano E, Bitchuette ME, Kappor BG (eds). Biology of subterranean fishes. (New Hampshire, USA: Science Publishers, **2010**, 169–280).

[R7] Q. Zheng, *A tentative exploration of circadian rhythm and activity intensity of three cavefishes in the Chinese endemic genus of Sinocyclocheilus*, The University of Chinese Academy of Sciences, **2017**.

[R8] F. Lei, M. Xu, Z. Ji, K. A. Rose, V. Zakirov, M. Bisset, *PLoS ONE*. **2022**, 17(7), e0270967.

[R9] A. Romero, Y. Zhao, X. Chen, *Environ. Biol. Fish.* **2009**, 86, 211–278.

[R10] H. Zheng, L. Xiu, J. Yang, *Environ. Biol. Fish.* **2013**, 96, 747–751.

[R11] Z. Ma, H. Herzog, Y. Jiang, Y. Zhao, D. Zhang, *Integr. Zool.* **2020**, 15, 314-328.
